# *Nolz1 *promotes striatal neurogenesis through the regulation of retinoic acid signaling

**DOI:** 10.1186/1749-8104-5-21

**Published:** 2010-08-24

**Authors:** Noelia Urbán, Raquel Martín-Ibáñez, Cristina Herranz, Miriam Esgleas, Empar Crespo, Monica Pardo, Ivan Crespo-Enríquez, Héctor R Méndez-Gómez, Ronald Waclaw, Christina Chatzi, Susana Álvarez, Rosana Álvarez, Gregg Duester, Kenneth Campbell, Angel R de Lera, Carlos Vicario-Abejón, Salvador Martinez, Jordi Alberch, Josep M Canals

**Affiliations:** 1 Department of Cell Biology, Immunology and Neuroscience, Faculty of Medicine, IDIBAPS, Universitat de Barcelona, C/Casanova 143, 08036 Barcelona, Spain; 2 Centro de Investigación Biomédica en Red sobre Enfermedades Neurodegenerativas (CIBERNED), Spain; 3 Department of Molecular Neurobiology, National Institute for Medical Research, The Ridgeway, Mill Hill, London NW7 1AA, UK; 4 Cell Therapy Program, Faculty of Medicine, Universitat de Barcelona, C/Casanova 143, 08036 Barcelona, Spain; 5 Alicante Neuroscience Institute, Miguel Hernandez University, Consejo Superior de Investigaciones Científicas (CSIC), 03550 San Juan de Alicante, Spain; 6 Departamento de Neurobiología Molecular, Celular y del Desarrollo, Instituto Cajal, Consejo Superior de Investigaciones Científicas (CSIC), C/Doctor Arce 37, 28002 Madrid, Spain; 7 Division of Developmental Biology, Children's Hospital Research Foundation, 3333 Burnet Avenue, Cincinnati, OH 45229, USA; 8 Development and Aging Program, Sanford-Burnham Medical Research Institution, 10901 North Torrey Pines Road, La Jolla, CA 92093, USA; 9 Departamento de Química Orgánica, Universidade de Vigo, Lagoas-Marcosende s/n, 36310 Vigo, Spain

## Abstract

**Background:**

*Nolz1 *is a zinc finger transcription factor whose expression is enriched in the lateral ganglionic eminence (LGE), although its function is still unknown.

**Results:**

Here we analyze the role of *Nolz1 *during LGE development. We show that *Nolz1 *expression is high in proliferating neural progenitor cells (NPCs) of the LGE subventricular zone. In addition, low levels of *Nolz1 *are detected in the mantle zone, as well as in the adult striatum. Similarly, *Nolz1 *is highly expressed in proliferating LGE-derived NPC cultures, but its levels rapidly decrease upon cell differentiation, pointing to a role of *Nolz1 *in the control of NPC proliferation and/or differentiation. In agreement with this hypothesis, we find that *Nolz1 *over-expression promotes cell cycle exit of NPCs in neurosphere cultures and negatively regulates proliferation in telencephalic organotypic cultures. Within LGE primary cultures, *Nolz1 *over-expression promotes the acquisition of a neuronal phenotype, since it increases the number of β-III tubulin (Tuj1)- and microtubule-associated protein (MAP)2-positive neurons, and inhibits astrocyte generation and/or differentiation. Retinoic acid (RA) is one of the most important morphogens involved in striatal neurogenesis, and regulates *Nolz1 *expression in different systems. Here we show that *Nolz1 *also responds to this morphogen in E12.5 LGE-derived cell cultures. However, *Nolz1 *expression is not regulated by RA in E14.5 LGE-derived cell cultures, nor is it affected during LGE development in mouse models that present decreased RA levels. Interestingly, we find that *Gsx2*, which is necessary for normal RA signaling during LGE development, is also required for *Nolz1 *expression, which is lost in *Gsx2 *knockout mice. These findings suggest that *Nolz1 *might act downstream of *Gsx2 *to regulate RA-induced neurogenesis. Keeping with this hypothesis, we show that *Nolz1 *induces the selective expression of the RA receptor (RAR)β without altering RARα or RARγ. In addition, *Nozl1 *over-expression increases RA signaling since it stimulates the RA response element. This RA signaling is essential for *Nolz1*-induced neurogenesis, which is impaired in a RA-free environment or in the presence of a RAR inverse agonist. It has been proposed that *Drosophila Gsx2 *and *Nolz1 *homologues could cooperate with the transcriptional co-repressors Groucho-TLE to regulate cell proliferation. In agreement with this view, we show that *Nolz1 *could act in collaboration with TLE-4, as they are expressed at the same time in NPC cultures and during mouse development.

**Conclusions:**

*Nolz1 *promotes RA signaling in the LGE, contributing to the striatal neurogenesis during development.

## Background

During the first stages of striatal development, neurons arise from the ventricular zone (VZ), which is mainly composed of neuroepithelial cells [1,2]. Around embryonic day (E)11.5, these cells divide asymmetrically; giving rise to radial glial cells and neural progenitor cells (NPCs). Radial glial cells have extensions that contact with the ventricular lumen and with more differentiated inner zones of the developing striatum, but their cell bodies remain in the VZ [3-5]. In contrast, NPCs leave the VZ to proliferate and generate the so-called subventricular zone (SVZ). Within the dorsal telencephalon, the SVZ is a thin layer of cells, mainly formed of NPCs [4,6]. However, in the ventral telencephalon, it becomes a prominent structure that has been shown to be the main source of striatal neurons and glia. Striatal projecting neurons are born in the SVZ of the lateral ganglionic eminence (LGE) whereas the medial ganglionic eminence will give rise to cortical and striatal interneurons.

Retinoic acid (RA) is one of the morphogens that participates in the specification and differentiation of the intermediate position of the LGE in the telencephalon [7]. However, RA sources during telencephalon development are still unclear. Three different retinaldehyde dehydrogenases (Raldhs), the limiting enzymes for RA synthesis, have been described in the central nervous system. Two of them, Raldh2 and Raldh3, are expressed in the otic vesicles and frontonasal ectoderm, respectively, as early as E8.5 [8-10]. Thus, it is unlikely that RA from these sources could reach the intermediate telencephalon. Around E12.5, Raldh3 expression appears in the LGE, providing the first known source of RA in the striatum [9,11]. During this period, the expression of the RA receptors (RARs) RARα and RARβ is high in the ventral telencephalon and it has been shown that RARβ stimulation mediates gene regulation in the developing telencephalon, particularly on striatal neuronal populations [12] where 95% of neurons are GABAergic [13]. In addition, RA increases the number of GABAergic neurons in differentiating mouse embryonic stem cells through the regulation of RARβ [14,15]. Some transcription factors are of great importance for RA signaling in the LGE. Among these is the homeobox transcription factor *Gsx2*, which is essential for correct striatal development [16,17] and for Raldh3 expression. Raldh3 levels are severely reduced or lost in mice deficient for *Gsx2 *or *Gsx1 *and *Gsx2 *[18].

To exert its effect during development, RA binds specific RARs and, thereafter, regulates the expression of some transcription factors that contain a RA response element (RARE) in their promoter. One of these transcription factors is Nolz1, which is induced by RA in the PC12 neural cell line and during developing chick spinal cord [19,20]. Nolz1 is a member of the NocA-Elbow (*elB*)-Tlp (NET) family of transcription factors, which are involved in patterning and differentiation during development in all studied species [21-24]. In zebrafish, two members of the NET family have been identified, *nlz1 *and *nlz2*, and both have been described to be essential for boundary formation in the rombencephalon [23,24]. *Nlz2 *is also expressed in more anterior structures, including the telencephalic vesicles, where it is supposed to play similar roles in regionalization [25]. A *Nlz2 *homolog in rodents was called *Nolz1 *(also known as *Zfp503*), which is expressed during nervous system development in several regions, including the hypothalamus and spinal cord. However, its highest expression is localized in the ventral LGE (vLGE) [19,26]. Of note, *Nolz1 *is totally absent in close telencephalic structures such as the medial ganglionic eminence and the pallidum, including the dorsal LGE (dLGE) [19], suggesting a highly specific striatal function. However, this family of transcription factors cannot directly interact with DNA, indicating that they need to interact with other transcription factors. In *Drosophila*, it has been shown that elbow B (elB) interacts with Groucho (Gro) proteins, forming large complexes of proteins that act as transcriptional co-repressors [27].

Despite its characterized pattern of expression, the function of *Nolz1 *during telencephalic development has not been analyzed yet. Here we studied the role of *Nolz1 *during vLGE development and the relationship between *Nolz1 *and the RA pathway within the LGE. Our results show that *Nolz1 *induces cell cycle exit and promotes neuronal differentiation. In addition, we also demonstrate that *Nolz1 *expression is temporarily regulated by RA during early striatal development. Finally, we show that *Nolz1 *contributes to neuronal differentiation through the increase of RA signaling.

## Results

### *Nolz1 *expression is regulated during striatal development

**Figure 1:**
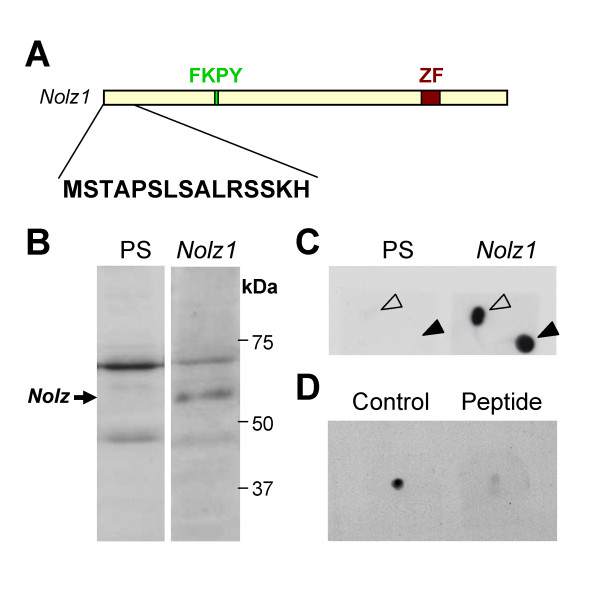
**Characterization of the anti-Nolz1 polyclonal antibody. (A) **Representation of Nolz1 showing the peptide against which the polyclonal antibody was raised. FKPY, Groucho consensus binding site; ZF, zinc-finger domain. **(B) **Western blots of NPC protein extracts, showing endogenous Nolz1 expression. Membranes were incubated with a rabbit serum obtained before (pre-serum; PS) or after (Nolz1) immunization. The specific band corresponding to Nolz1 (63 kDa; arrow) is visible only with the anti-Nolz1 antibody. **(C) **Dot-blot against Nolz1 pure peptide (upper drop; open arrowheads) or Keyhole limpet hemocyanin (KLH)-conjugated Nolz1 peptide (lower drop; closed arrowheads) incubated with rabbit serum before (pre-serum; PS) and after (Nolz1) immunization, showing Nolz1 antibody specificity. **(D) **Nolz1 pure peptide dots immuno-blotted with the anti-Nolz1 polyclonal antibody pre-incubated in the presence (Peptide) or in the absence (Control) of Nolz1 peptide.

**Figure 2:**
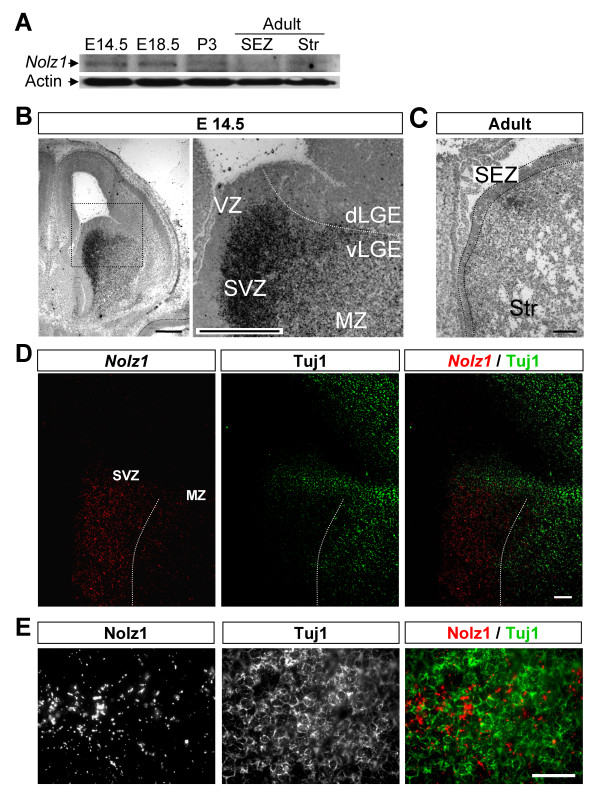
**The pattern of Nolz1 protein expression resembles that of *Nolz1 *mRNA in the LGE. (A) **Western blot analysis of striatal samples from different developmental stages showing high levels of Nolz1 protein at embryonic stages, which decrease during postnatal development. Within adult mice, Nolz1 expression remains at low levels in the striatum (Str) but is not detectable in the subependymal zone (SEZ). **(B) ***In situ *hybridization shows high levels of *Nolz1 *mRNA in the SVZ of the E14.5 vLGE. Note that the levels of *Nolz1 *mRNA decrease in the mantle zone (MZ). Scale bars: 600 μm. **(C) **Low levels of *Nolz1 *mRNA are also detected in the adult striatum but not in the subependymal zone. Scale bar: 150 μm. **(D) **Double *in situ *immunohistochemistry shows that within the MZ, Nolz1 expression is located in β-III tubulin (Tuj-1)-positive cells. Scale bar: 150 μm. **(E) **High magnification of (D). Scale bar: 60 μm.

In order to analyze whether the temporal expression pattern of Nolz1 protein resembles that of *Nolz1 *mRNA, a polyclonal antibody was raised against 12 amino acids of the amino terminus of the Nolz1 protein sequence (Figure 1A). Western blotting of NPC lysates showed that the antibody recognizes a unique specific band when compared to the pre-serum blotted membrane (Figure 1B). In addition, dot blot analyses demonstrated the specificity of the serum (Figure 1C), which could be blocked by the competitive incubation of the antibody with the Nolz1 peptide (Figure 1D). Using this Nolz1 polyclonal antibody, we performed western blot analysis in striatal samples from E14.5, E18.5, postnatal day (P)3 and adult mice. Our results showed that Nolz1 levels are high at embryonic stages, being down-regulated postnatally (Figure 2A). Within the adult, low levels of Nolz1 protein remained in the striatum while no expression was detected in the subependymal zone (Figure 2A). *In situ *hybridization for *Nolz1 *showed high levels of mRNA in the SVZ of the vLGE at E14.5 (Figure 2B). No signal was detected in the dLGE (Figure 2B), which gives rise to olfactory bulb interneurons during development [28]. Low levels of *Nolz1 *mRNA were also detected in the mantle zone (MZ; Figure 2B,D), where it partially co-localized with Tuj1-positive neurons (Figure 2D,E). In the adult brain, similar to the protein results, *Nolz1 *mRNA was not detected in the subependymal zone, although faint expression could still be detected in the striatum by *in situ *hybridization (Figure 2C).

### *Nolz1 *regulates NPC homeostasis in the LGE

**Figure 3:**
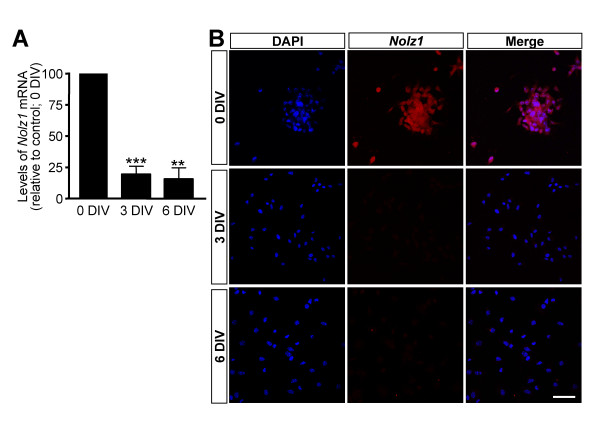
***Nolz1 *is highly expressed in proliferating NPCs and down-regulated during differentiation. (A) ***Nolz1 *mRNA expression is down-regulated during NPC differentiation. NPC samples from different stages of differentiation (0, 3 and 6 DIV) were analyzed by RT-PCR to study the expression pattern of *Nolz1*. The results are expressed as the percentage of the expression levels at 0 DIV, considered as 100%, and represent the mean from at least three independent samples at each condition. Error bars represent the standard error of the mean. Statistical analysis was performed with one-way ANOVA, followed by the Bonferroni *post-hoc *test. **P < 0.01, ***P < 0.001 relative to 0 DIV. **(B) **Nolz1 fluorescent immunocytochemistry was performed on NPC samples at different stages of differentiation (0, 3 and 6 DIV). Many Nolz1-positive cells were detected in proliferating neurospheres while the intensity and number of positive cells clearly decreased during differentiation. Scale bar: 50 μm.

The high levels of *Nolz1 *expression in the SVZ of the vLGE suggest it has a role in the regulation of NPC homeostasis. Thus, to analyze the function of *Nolz1 *in NPCs, we generated neurospheres from the LGE of E14.5 mice. *Nolz1 *expression was analyzed in proliferating cells and at 3 and 6 days *in vitro *(DIV) after the induction of neurosphere differentiation (Figure 3A,B). We observed that *Nolz1 *expression levels were high in non-differentiated cells, while its expression decreased during the differentiation process for both mRNA (Figure 3A) and protein (Figure 3B). These results were coincident with the expression pattern of *Nolz1 in vivo*, which mainly corresponded to the NPC-containing SVZ (Figure 2B).

**Figure 4:**
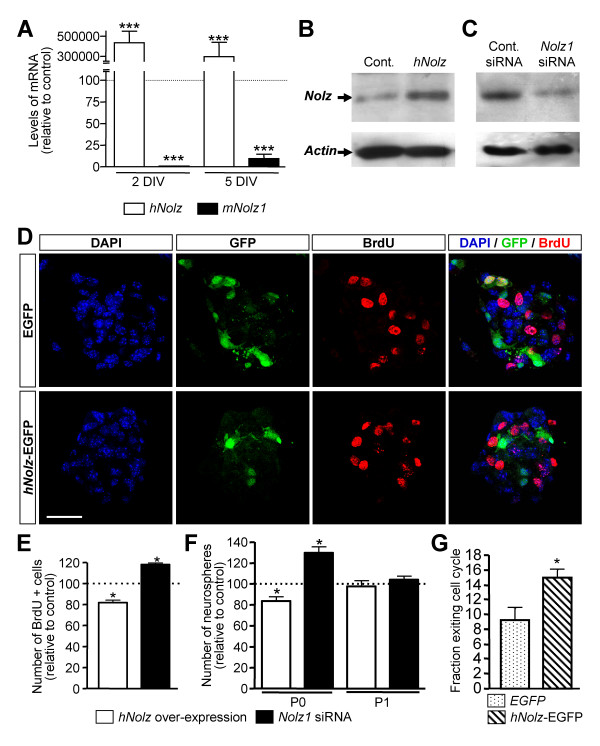
***Nolz1 *regulates proliferation and self-renewal of NPCs. (A) ***hNolz *over-expression produces a down-regulation of endogenous mouse *Nolz1 *(*mNolz1*) at 2 and 5 DIV after transfection with *hNolz1*-DsRED. Values are expressed as the mean percentage of control transfection (DsRED alone), considered as 100%. **(B) **Western blot of NPC protein extracts 5 DIV after transfection with DsRED control (Cont.) or hNolz-DsRED (hNolz), showing an increase in Nolz after *hNolz *transfection. **(C) **Western blot of NPC protein extracts 2 DIV after transfection of *Nolz1 *small interfering RNA (siRNA) or control siRNA. The expression of *Nolz1 *decreases about 82% in the cultures transfected with the *Nolz1 *siRNA relative to those transfected with the control siRNA. **(D,E) **Over-expression of *hNolz *in NPCs reduces the number of bromodeoxyuridine (BrdU)-positive cells, whereas *Nolz1 *siRNA transfection causes an increase in the number of BrdU-positive cells. Results are expressed as the relative number of BrdU-positive cells, standardized to their respective control, considered 100% (dotted line). **(F) ***Nolz1 *over-expression results in a decrease in neurosphere generation, while *Nolz1 *silencing results in an increase in the number of neurospheres just after transfection (P0, passage 0). When cells are dissociated and plated again (P1, passage 1), no differences were observed in any condition. Results are expressed as the percentage of counted neurospheres with respect to their respective control, considered 100% (dotted line). **(G) **Cell-cycle exit index was analyzed as the percentage of BrdU+/Ki67- cells with respect to the total number of BrdU-positive cells after a 3-DIV pulse label. *hNolz *induces a significant increase in the number of cells that leave the cell-cycle with respect to enhanced green fluorescent protein (EGFP)-over-expressing cells. The results in each graph represent the mean ± standard error of the mean from at least three independent samples at each condition. Statistical analysis was performed with the Student's *t*-test. *P < 0.05, ***P < 0.001 relative to the respective controls.

We next analyzed whether *Nolz1 *regulates NPC proliferation. Over-expression of human *Nolz *(*hNolz*) in NPCs led to high *hNolz *mRNA levels but a significant decrease in endogenous mouse *Nolz1 *expression (Figure 4A), suggesting tight regulation of mouse *Nolz1 *levels. When we analyzed Nolz protein levels with a Nolz1 antibody that detects mouse and human isoforms, we found a net increase in Nolz protein levels with respect to control transfected cells (Figure 4B). *hNolz*-over-expressing neurospheres showed a 19.5% reduction in the number of proliferating bromodeoxyuridine (BrdU)-positive NPCs with respect to control transfected cells (Figure 4D,E). Double immunocytochemistry showed that most, but not all, of the *hNolz*-over-expressing cells were negative for BrdU (Figure 4D). To verify that the reduction in BrdU-positive cells was due to cell cycle exit and not to variations in its duration, we measured the cell cycle time of transfected NPCs as described elsewhere [29] and did not find any difference between control and hNolz-over-expressing neurospheres (DsRED, 25.29 h; hNolz-DsRED, 24.88 h). In addition, we analyzed the index of cell cycle exit as described previously by Chenn and Walsh [30]. This demonstrated that *hNolz *over-expression produces a significant increase of 162% in the cell cycle exit index (Figure 4G). Since the reduction in the number of BrdU-positive NPCs may also be due to cell death, we also counted the number of apoptotic nuclei of *hNolz*-over-expressing NPCs with respect to control enhanced green fluorescent protein (EGFP)-transfected NPCs. No cell death was observed in any condition (data not shown).

In addition, the effect of *Nolz1 *silencing was also analyzed by using a cocktail of three different *Nolz1 *small interfering RNAs (siRNAs) at a concentration of 2 μM, which led to 60% inhibition of *Nolz1 *mRNA (data not shown) and 82% inhibition of Nolz1 protein expression 2 DIV after transfection (Figure 4C). As expected, transfection of *Nolz1 *siRNA increased the number of BrdU-positive NPCs (by 18.5%) in the neurosphere cultures with respect to scrambled negative siRNA control transfection (Figure 4E). Taken together, these findings demonstrate that deregulation of *Nolz1 *altered the proliferating capacity of NPCs, suggesting that *Nolz1 *promotes cell cycle exit. Next, we studied the capacity of NPCs to form new neurospheres 5 DIV after *hNolz *over-expression or transfection of *Nolz1 *siRNA (Figure 4F). *hNolz *negatively regulated NPC self-renewal, as shown by the decrease in the number of new neurospheres formed after *hNolz *over-expression and the corresponding increase observed after *Nolz1 *siRNA transfection (Figure 4E, P0). When cells were dissociated and plated again (P1), no differences were observed in any condition studied (Figure 4E). These findings indicate a transient effect of *Nolz1 *and that variation of the levels of this transcription factor does not permanently affect NPC populations since *hNolz *over-expression or *Nolz1 *siRNA was lost after the first passage.

**Figure 5:**
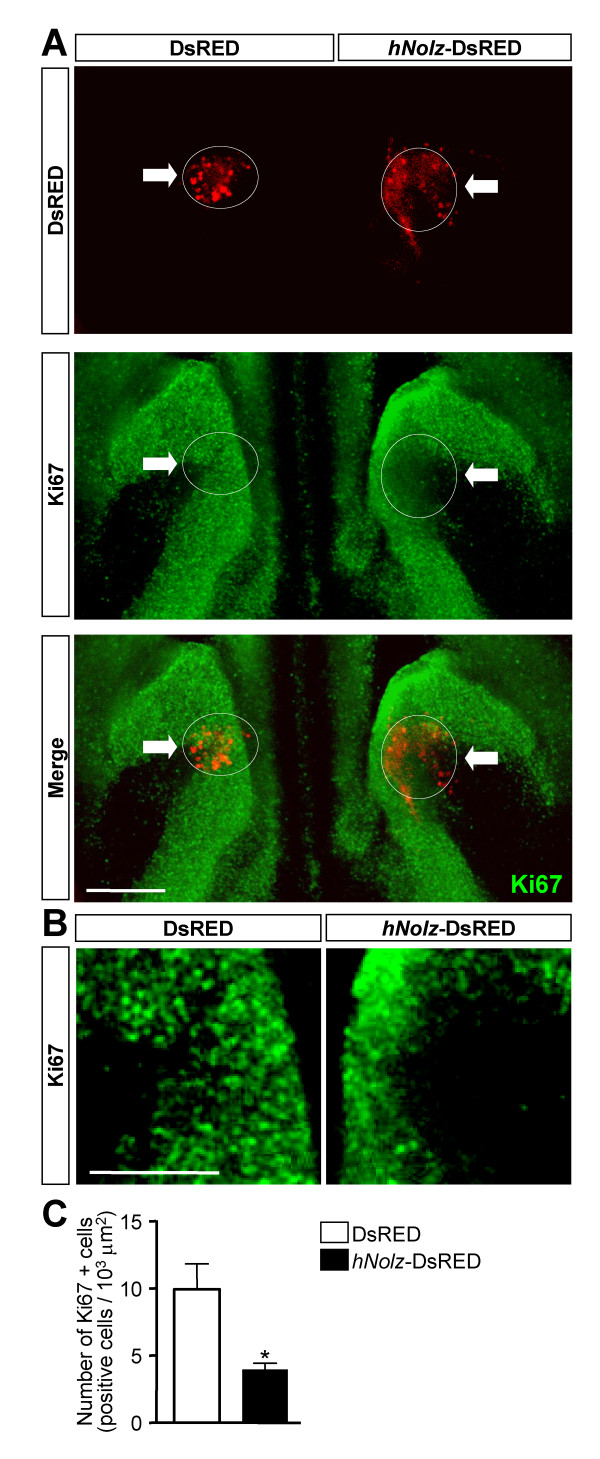
***Nolz *decreases NPC proliferation in the SVZ of LGE organotypic cultures. (A) **Organotypic cultures from E15.5 LGE were electroporated in the VZ/SVZ with *hNolz *(*hNolz*-DsRED) or the control vector (DsRED) and stained for Ki67 (white arrows indicate the fluorescent DsRED-expressing cells). Over-expression of *hNolz *produces a reduction in cell proliferation in the SVZ as shown by the reduction in Ki67-positive cells in the electroporated area. Scale bar: 600 μm. **(B) **High magnification of the electroporated area showing the decrease in Ki67-positive cells in the SVZ of the LGE. Scale bar: 300 μm. **(C) **Quantification of the number of Ki67-positive cells in the transfected area. The results represent the mean ± standard error of the mean from four independent experiments. Statistical analysis was performed with the Student's *t*-test; *P < 0.05.

To further confirm the role of *Nolz1 *in the regulation of NPC proliferation, we next electroporated *hNolz *into LGE-derived organotypic cultures from E15.5 embryos and analyzed the number of Ki67-positive cells (Figure 5). Two days after electroporation, *hNolz *over-expression reduced the proliferation of NPCs at the SVZ as indicated by the dramatic decrease in the number of Ki67-positive cells with respect to the control electroporated side (Figure 5B).

### *Nolz1 *over-expression promotes the acquisition of a neuronal phenotype in LGE primary cultures

**Figure 6:**
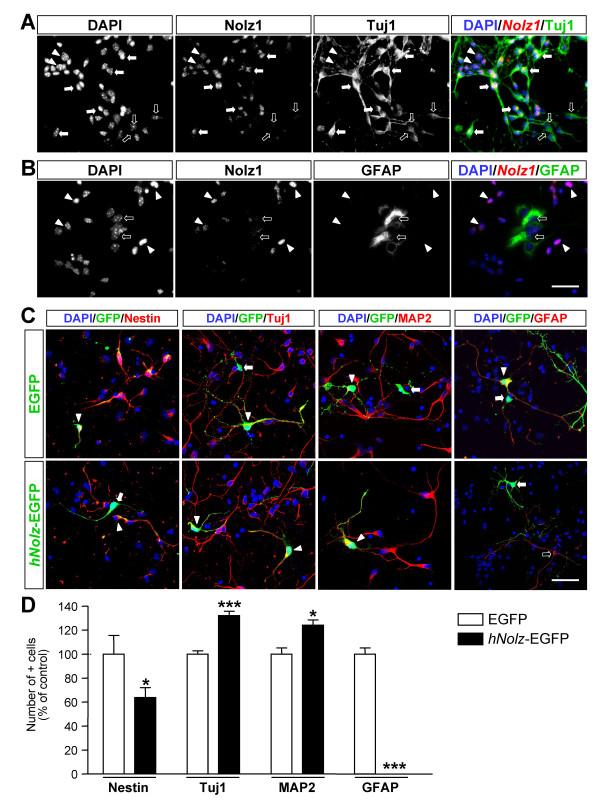
***Nolz1 *acts as a neurogenic factor in primary LGE cultures. (A,B) **Double fluorescent immunocytochemistry performed on primary E14.5 LGE cultures. Nolz1 is expressed in cells positive for the early neuronal marker Tuj1 (A). In contrast, Nolz1 is not detected in cells expressing the astroglial marker glial fibrillary acidic protein (GFAP) (B). White arrows show double positive cells, open arrows show single stained cells for Tuj1 or GFAP, and white arrowheads show single stained cells for Nolz1. Scale bar: 50 μm. **(C,D) **Over-expression of *hNolz *in LGE primary cultures increased the number of neurons at the expense of glial fates as shown by the increase in the number of Tuj1- or MAP2-positive cells and the complete blockade of astroglial cells. Note that all cells over-expressing *hNolz *are negative for the GFAP marker (C). (C) White arrows show single stained cells for Nolz1, white arrowheads show double positive cells and open arrows show single GFAP-positive cells. Scale bar: 50 μm. **(D) **The results represent the mean ± standard error of the mean from at least three independent samples at each condition. Statistical analysis was performed with the Student's *t*-test. *P < 0.05, ***P < 0.001 relative to EGFP control.

Reduced proliferation of NPCs could be indicative of neural differentiation. To test this possibility, we first analyzed whether *Nolz1 *was expressed in differentiated post-mitotic cells in primary cultures derived from E14.5 LGEs (Figure 6A,B). Double immunocytochemistry against Nolz1 and the neural markers Tuj1 or glial fibrillary acidic protein (GFAP) showed that most of the Nolz1-positive cells were positive for the neuronal marker Tuj1, while we could not find any overlap between Nolz1 and the astroglial marker GFAP (Figure 6A,B). We also analyzed the role of *Nolz1 *on neural differentiation by transfecting LGE primary cultures with plasmids that express *hNolz*-EGFP or EGFP alone as control (Figure 6C,D). Five days after transfection, we performed double immunocytochemistry for GFP and the neural precursor gene nestin, the neuronal markers Tuj1 and microtubule-associated protein (MAP)2 or the astroglial marker GFAP. *hNolz*-over-expressing cells mainly colocalized with the neuronal markers Tuj1 and MAP2, although some double GFP-nestin stained cells were also observed (Figure 6C). In contrast, no astroglial cell markers were seen in *hNolz *transfected cells, as shown by the lack of colocalization between GFP and GFAP (Figure 6C). Interestingly, the quantification of the double positive cells in *hNolz *(*hNolz*-EGFP) versus control transfected cells (EGFP) demonstrated that *hNolz *promotes a neuronal phenotype (Figure 6D). *hNolz *over-expression increased the number of both Tuj1- and MAP2-positive neurons, which was accompanied by a reduction in the number of nestin-positive cells (Figure 6D). In addition, no *hNolz*-over-expressing cells were GFAP-positive astroglia (Figure 6D).

### *Nolz1 *expression is downstream of *Gsx2 *and its levels are temporarily regulated by RA during striatal development

**Figure 7:**
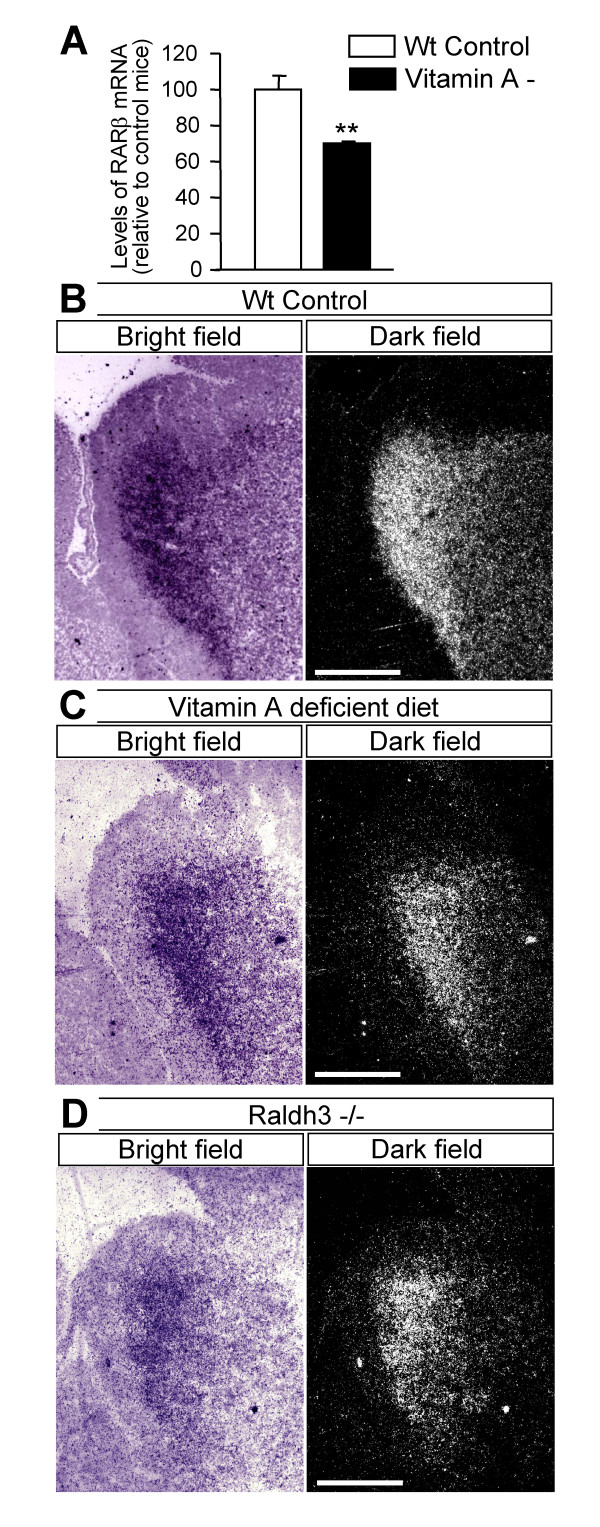
***Nolz1 *expression does not depend on RA signaling in the LGE *in vivo*. (A) **Embryos developed under a vitamin A-deficient diet (vVitamin A -) had reduced levels of RARβ expression compared to wild-type control mice (WT Control). Values are normalized to control (regular diet), considered as 100%, and expressed as the mean of four independent samples for each condition. Error bars represent the standard error of the mean. Statistical analysis was performed with the Student's *t*-test. **P < 0.01 relative to control. **(B) ***In situ *hybridization for *Nolz1 *performed in regular fed wild-type E14.5 embryos. **(C) ***In situ *hybridization at E14.5 shows normal *Nolz1 *expression levels. **(D) **Similarly, Raldh3^-/- ^embryos at E14.5 show normal *Nolz1 *expression. Scale bars: 600 μm.

To determine whether RA is needed for *Nolz1 *expression in striatal cells during development, pregnant mice were fed with a vitamin A (retinol)-deficient diet, which results in a general decrease of RA blood levels [31]. In these animals the levels of *RARβ *were partially reduced (Figure 7A), indicating a decrease in RA levels since it is well known that this receptor is regulated by RA signals. Analysis of *Nolz1 *expression in E14.5 embryos developed under these vitamin A-deficient levels did not show any difference with respect to expression in regular fed wild-type control embryos (Figure 7B,C). To further confirm that the lack of RA does not affect *Nolz1 *expression, we analyzed the levels of *Nolz1 *expression in Raldh3^-/- ^embryos (Figure 7D), which have completely lost RA activity [11]. *Nolz1 *expression was not affected by the absence of Raldh3 in the LGE at E14.5 (Figure 7D), further corroborating the independence of *Nolz1 *expression from RA signaling in the vLGE at this developmental stage.

**Figure 8:**
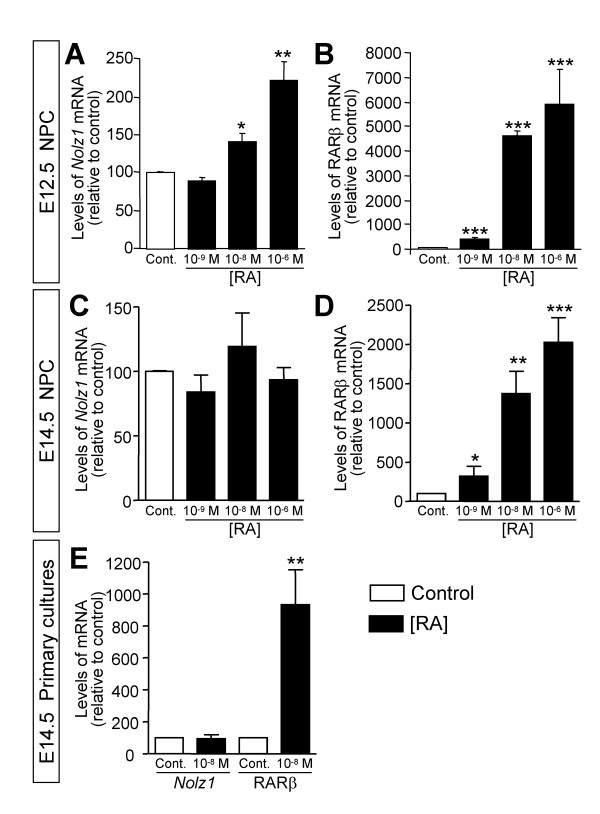
**Striatal cultures are temporarily competent to regulate *Nolz1 *expression in response to RA. **NPC and primary cultures were treated with RA and the expression of *Nolz1 *and *RARβ *were analyzed by Q-PCR. **(A,B) **RA increases the expression of *Nolz1 *and *RARβ *in a dose-dependent manner (0, 10^-9^, 10^-8 ^and 10^-6 ^M) in E12.5-derived neurospheres. **(C,D) **Within E14.5-derived neurospheres, RA increases the expression of *RARβ *in a dose-dependent manner (D) but it does not affect *Nolz1 *mRNA levels (C). **(E) **RA increases the levels of *RARβ *without affecting *Nolz1 *mRNA levels in LGE primary cultures treated with 10^-8 ^M RA during 3 DIV. The results represent the mean ± standard error of the mean from at least three independent samples at each condition. Statistical analysis was performed with one-way ANOVA, followed by the Bonferroni *post-hoc *test (A-D) or with the Student's *t*-test (E). *P < 0.05, **P < 0.01, ***P < 0.001 relative to control.

**Figure 9:**
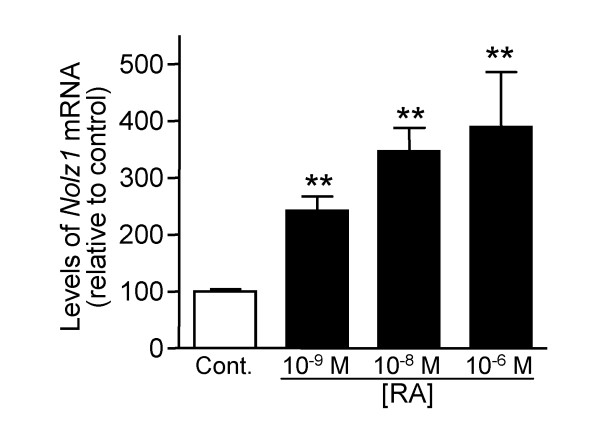
***Nolz1 *expression is induced by RA in embryoid bodies derived from mouse embryonic stem cells. **Embryoid bodies were treated with various concentrations of RA (0, 10^-9^, 10^-8 ^and 10^-6 ^M) and 4 DIV later *Nolz1 *expression levels were analyzed by Q-PCR. Values are normalized to control (without RA), considered as 100%, and expressed as the mean from at least three independent samples at each stage studied. Error bars represent the standard error of the mean. Statistical analysis was performed with one-way ANOVA, followed by the Bonferroni *post-hoc *test. **P < 0.01 relative to control.

To analyze whether *Nolz1 *expression levels were regulated by RA *in vitro*, LGE-derived neurospheres were treated with increasing concentrations of RA during 3 DIV and the levels of *Nolz1 *were analyzed by quantitative PCR (Q-PCR). *Nolz1 *expression was increased by RA in E12.5-derived NPC cultures (Figure 8A). However, it was not affected in E14.5-derived NPC cultures (Figure 8C). As a well known RA-induced control gene, we analyzed *RARβ*, which was clearly upregulated in a dose-dependent manner at both stages (Figure 8B,D). Coincidentally, *Nolz1 *expression did not change in E14.5 LGE primary cultures treated with RA, although *RARβ *was also increased in these cultures (Figure 8E). Therefore, striatal cultures were competent to increase *Nolz1 *expression in response to RA treatment at E12.5 but not at later stages. In agreement with these results, when we treated mouse embryonic stem cells with RA, *Nolz1 *expression was upregulated in a dose-dependent manner (Figure 9), supporting that *Nolz1 *competence to RA signaling depends on early developmental stages.

**Figure 10:**
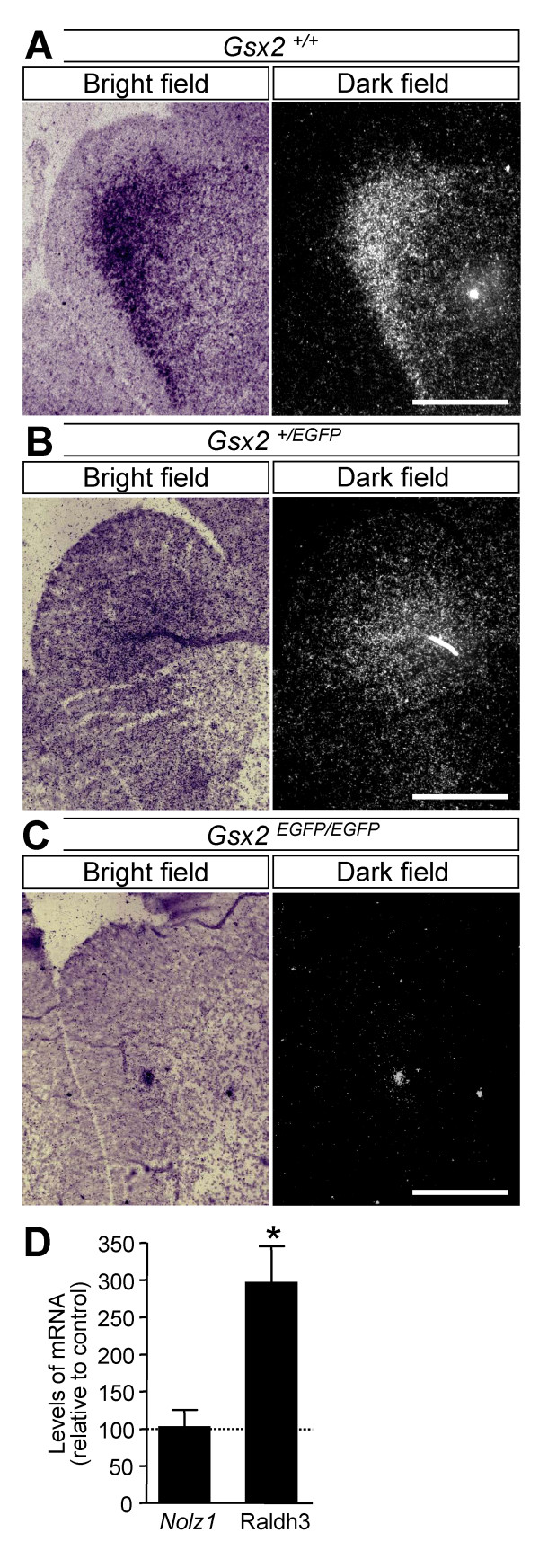
***Gsx2 *is a permissive factor for *Nolz1 *expression. (A-C) ***Nolz1 *mRNA expression is detected in E14.5 wild-type mice (*Gsx2*^*+/+*^; A) and *Gsx2*^*+/EGFP *^mice (B), but totally disappears in the vLGE of *Gsx2*^*EGFP/EGFP *^mice (C). Scale bars: 600 μm. **(D) ***Gsx2 *over-expression in NPCs does not modify *Nolz1 *mRNA levels, although it increases *Raldh3 *mRNA expression. The results represent the mean ± standard error of the mean from at least three independent samples at each condition. Statistical analysis was performed with the Student's *t*-test. *P < 0.05, relative to control.

Since *Gsx2 *is essential for the correct expression of several RA-dependent genes, such as *Raldh3*, in the LGE [18], we also examined the expression of *Nolz1 *in *Gsx2 *knockout mice. *Nolz1 *expression was analyzed by *in situ *hybridization in knockout mice carrying a single (*Gsx2*^+/EGFP^) or double substitution of the *Gsx2 *gene for EGFP (*Gsx2*^EGFP/EGFP^) [32]. Low levels of *Nolz1 *expression remained in the *Gsx2 *heterozygous mouse vLGE at E14.5 (Figure 10A,B), but no signal was detected in the *Gsx2*^EGFP/EGFP ^mice (Figure 10C), indicating that *Gsx2 *is critical for *Nolz1 *expression. On the other hand, over-expression of *Gsx2 *in NPCs did not lead to an increase in *Nolz1 *mRNA levels, while it enhanced the levels of *Raldh3 *mRNA (Figure 10D), which has been shown to be regulated by *Gsx2 *[18]. These findings indicate that *Gsx2 *is essential but not sufficient to induce *Nolz1 *expression.

### RA signaling is necessary to induce *Nolz1*-dependent neurogenesis

**Figure 11:**
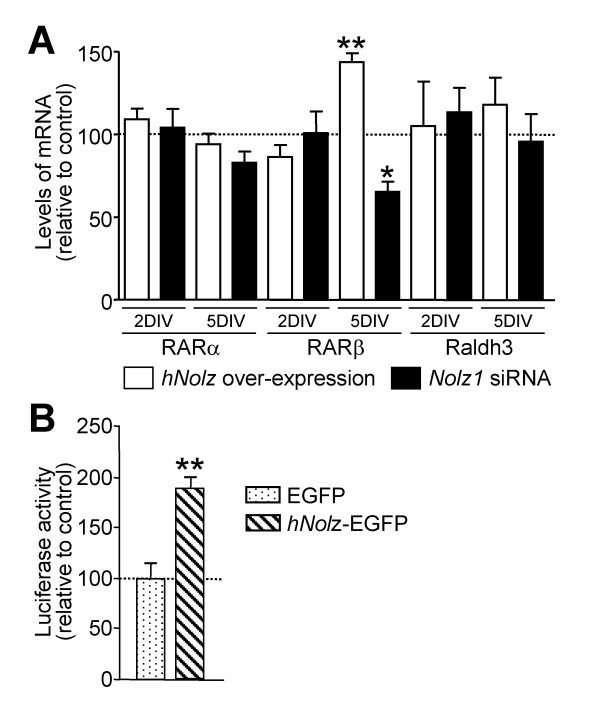
***Nolz1 *increases RARβ expression and induces RA signaling. (A) **RT-PCR analysis of *RARα*, *RARβ *and *Raldh3 *mRNA at 2 and 5 DIV after transfection of *hNolz *(*hNolz *over-expression) or *Nolz1 *siRNA. The results are expressed as the mean ± standard error of the mean from at least three independent samples for each condition, and normalized to the respective control transfection (DsREd or control siRNA), considered as 100% (dotted line). **(B) **RARE luciferase reporter assay demonstrates that *Nolz1 *over-expression increases RA signaling in primary striatal cultures. The results are expressed as the mean ± standard error of the mean from four independent experiments, and normalized to the respective control transfection (EGFP), considered as 100%. *P < 0.05, **P < 0.01, relative to control.

**Figure 12:**
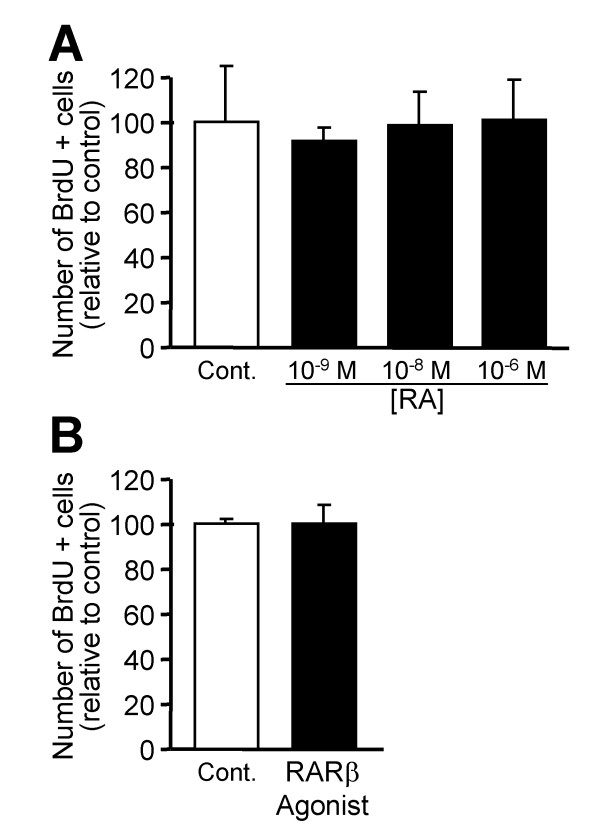
**RA does not affect the proliferation of NPCs in culture. (A) **NPCs grown as neurospheres were treated with various concentrations of RA (0, 10^-9^, 10^-8 ^and 10^-6 ^M) and after 3 DIV the number of BrdU-positive cells was analyzed. RA treatment did not have any effect on NPC proliferation at any of the concentrations studied. **(B) **The same result was observed when NPC cultures were treated with a RARβ-specific agonist (10^-8 ^M). Values are normalized to control (not treated with RA or RARβ agonist), considered as 100%, and expressed as the mean ± standard error of the mean from at least three independent samples for each condition.

**Figure 13:**
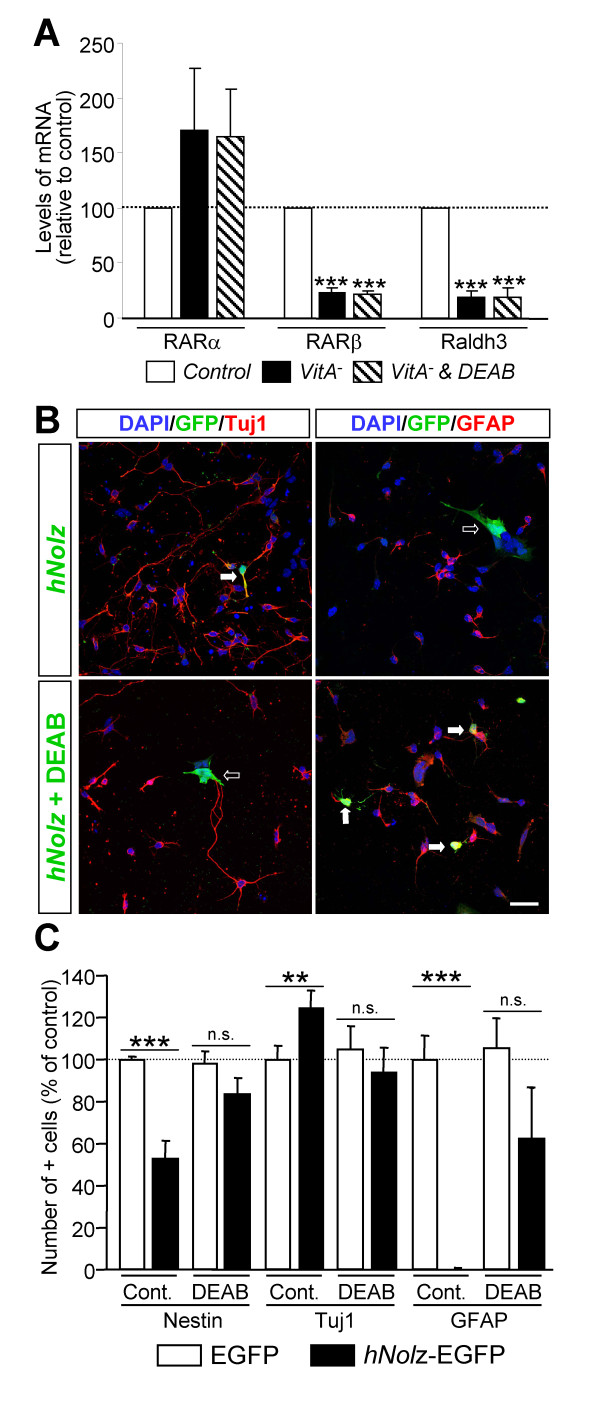
***Nolz1*-mediated neurogenesis depends on RA signaling. (A) **RT-PCR analysis of *RARα*, *RARβ *and *Raldh3 *mRNA expression in NPC cultures grown in normal conditions (control), in vitamin A-free medium (VitA^-^) and in vitamin A-free medium in the presence or DEAB (VitA^- ^& DEAB) demonstrates a decrease in *RARβ *and *Raldh3 *mRNA in the absence of RA. The results are expressed as the mean ± standard error of the mean from at least three independent samples for each condition, and normalized to the control medium, considered as 100%. Statistical analysis was performed with the Student's *t*-test. ***P < 0.001 relative to control. **(B,C) ***Nolz1 *does not induce neuronal differentiation in a RA-free environment. (B) Representative pictures of immunofluorescence for Tuj1 or GFAP after transfection of *hNolz *in the absence or presence of DEAB. White arrows show double positive cells, open arrows show single EGFP stained cells. Scale bar: 30 μm. **(C) **Treatment with 1 μM DEAB in a vitamin A-free medium (DEAB) impairs the increase of Tuj1 and the reduction of GFAP observed in *hNolz*-transfected primary striatal cultures in control conditions (Cont.). The results are expressed as the mean ± standard error of the mean from at least three independent samples for each condition, and normalized to the control medium, considered as 100%. Statistical analysis was performed with the Student's *t*-test (C). **P < 0.01, ***P < 0.001 relative to control. N.s., not significant.

**Figure 14:**
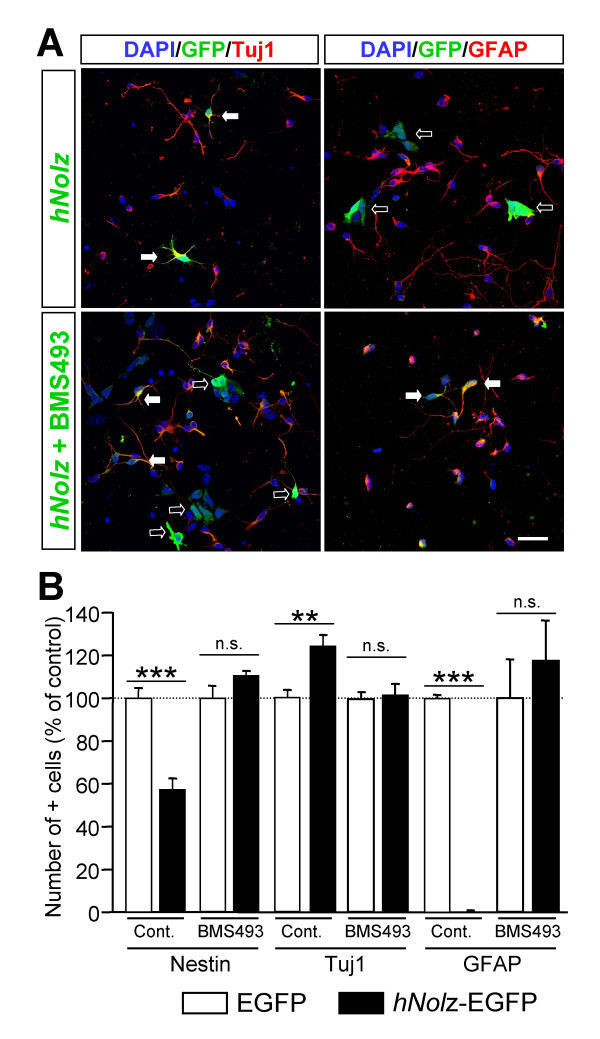
**RAR activity is essential for *Nolz1*-mediated neurogenesis. (A,B) ***Nolz1 *does not induce neuronal differentiation in the presence of a RAR inverse agonist. **(A) **Representative pictures of immunofluorescence for Tuj1 or GFAP after transfection of *hNolz *in the absence or presence of a RAR inverse agonist (BMS493). White arrows show double positive cells, open arrows show single EGFP stained cells. Scale bar: 30 μm. **(B) **The presence of the RAR inverse agonist (10^-8 ^M BMS493) impairs the increase of Tuj1 and the reduction of GFAP observed after *hNolz *transfection in primary striatal cultures (Cont.). The results are expressed as the mean ± standard error of the mean from at least three independent samples for each condition, and normalized to the transfection in standard culture conditions (Cont.), considered as 100%. Statistical analysis was performed with the Student's *t*-test. **P < 0.01, ***P < 0.001 relative to control.

**Figure 15:**
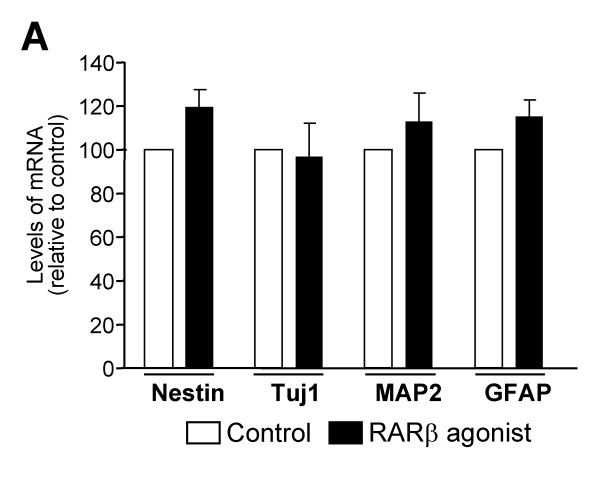
**RARβ stimulation is not sufficient to induce neurogenesis in primary striatal cultures. **RT-PCR analyses of neural markers in primary striatal cultures treated with an RARβ agonist (10^-8 ^M; BMS641) demonstrate that it does not modify striatal neurogenesis. The results are expressed as the mean ± standard error of the mean from at least three independent samples for each condition, and normalized to the control medium without the RARβ agonist, considered as 100%. Statistical analysis was performed with the Student's *t*-test.

The specific combination of RA receptors defines the competence of cells to respond to RA. Thus, we next analyzed whether *Nolz1 *expression regulates RARs. We measured *RARα*, *RARβ *and *RARγ *mRNA levels 2 and 5 DIV after *hNolz *over-expression or silencing with *Nolz1 *siRNA in NPCs (Figure 11A). We did not detect *RARγ *expression in control NPCs, or after transfection of *hNolz *or *Nolz1 *siRNA (data not shown). In contrast, both *RARα *and *RARβ *were highly expressed in NPCs. *RARα *levels were not modified in any condition studied whereas *RARβ *levels were increased 5 DIV after *hNolz *over-expression and reduced after *Nolz1 *silencing (Figure 11A). In addition, *hNolz *over-expression did not regulate the level of *Raldh3 *mRNA (Figure 11A). Similarly, the levels of *CRBP1 *and *Cyp26*, two other limiting proteins for RA metabolism, were not modified by *hNolz *overexpression (data not shown). These findings indicate that *Nolz1 *expression does not increase RA levels but changes the competence of cells to respond to RA. To further test this hypothesis, we analyzed whether *hNolz *over-expression could activate RA signaling using a Luciferase RARE-reporter assay. Three DIV after transfection, an increase in luciferase activity was observed (Figure 11B), demonstrating that this transcription factor leads to an increase in RA signaling. These results suggested that *Nolz1 *could induce the conversion of NPCs into neuronal cells through the regulation of RARβ-mediated signaling. Thus, we next analyzed whether *Nolz1 *mediates NPC cell cycle exit and promotes neuronal differentiation by this mechanism. Surprisingly, RA signaling does not seem to mediate *Nolz1*-regulated proliferation of NPCs, since under the same conditions that *hNolz *modified BrdU incorporation, treatment with RA (Figure 12A) or a RARβ-specific agonist (Figure 12B) did not affect neurosphere proliferation (Figure 12). Thus, we next analyzed whether RA signaling was necessary for *Nolz1*-induced neurogenesis. To this end, we transfected primary striatal cultures with *hNolz *in the absence of RA, since cells were cultured in a RA-free medium supplemented with 4-diethylaminobenzaldehyde (DEAB), a Raldh inhibitor [33]. Under these conditions, the levels of *RARβ *and *Raldh3 *were highly reduced (Figure 13A). Moreover, *hNolz *could not induce neuronal differentiation in the absence of RA, since *hNolz*-induced neurogenesis was lost in the presence of DEAB (Figure 13B,C). To further study whether the effect of *Nolz1 *in neurogenesis was not only dependent on RA but also on RARβ signaling, we next over-expressed *hNolz *in striatal primary cultures treated with a pan-RAR inverse agonist (BMS493, also named UVI2024, [34,35]). In these conditions, *hNolz *was also unable to induce an increase of Tuj1-positive cells and a reduction of nestin-positive precursors or GFAP-positive astroglia (Figure 14). Therefore, these results demonstrate that *Nolz1 *needs RA signaling through RARs to mediate striatal neurogenesis. However, the stimulation of RARβ with a specific agonist (BMS641, also named UVI2003, [36]) was not sufficient to induce changes in neural markers in striatal cultures (Figure 15).

### *Tle4 *and *Nolz1 *have parallel expression patterns in both the LGE *in vivo *and LGE-derived NPCs

**Figure 16:**
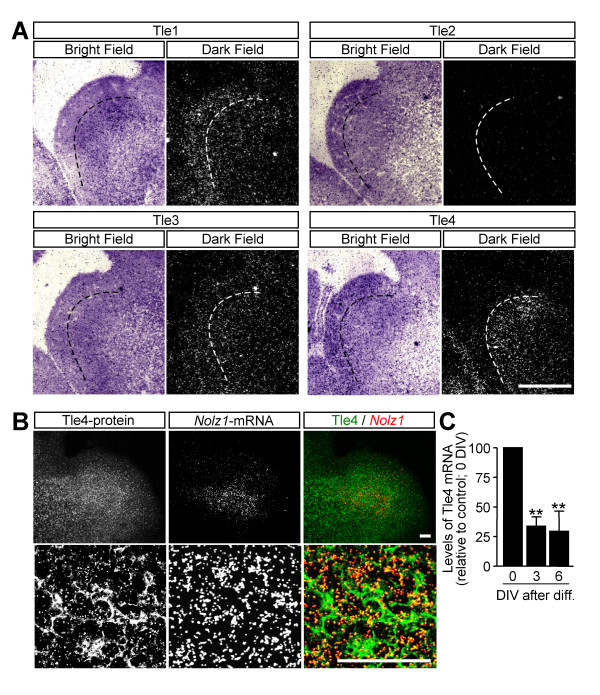
***Tle4 *and *Nolz1 *expression show similar patterns in the developing LGE and in NPCs. (A) ***In situ *hybridization study to analyze *Tle1*, *Tle2*, *Tle3 *and *Tle4 *mRNA levels in the LGE at E14.5. Dashed lines mark the VZ-SVZ boundary. **(B) **Simultaneous Tle4 immunohistochemistry and *Nolz1 in situ *hybridization show coincident expression patterns in the LGE at E14.5. **(C) **High levels of *Tle4 *mRNA are observed in proliferating NPCs (0 DIV), which decrease after differentiation (3 and 6 DIV). The results are expressed as the mean from at least three independent samples for each condition, and normalized to the proliferating NPCs (0 DIV), considered as 100%. Error bars represent the standard error of the mean. Statistical analysis was performed with one-way ANOVA, followed by the Bonferroni *post-hoc *test. **P < 0.01 relative to control. Scale bars: 600 μm (A); 100 μm (B).

Due to the ability of the NET family of transcription factors, which includes *Nolz1*, to bind to members of the Gro-TLE family of transcriptional repressors [22,20,24], we analyzed the expression of several members of the Gro-TLE family in the LGE at E14.5 by *in situ *hybridization (Figure 16). We did not detect any *Tle2 *expression, and *Tle3 *mRNA was slightly but broadly expressed through the telencephalon (Figure 16A). *Tle1 *and *Tle4*, in contrast, were specifically regulated in the different proliferative zones of the LGE (Figure 16A). *Tle1 *expression was maximal in the VZ, and decreased in the SVZ and MZ (Figure 16A). In contrast, *Tle4 *was not expressed in the VZ and its levels were high in the SVZ, extending to the MZ but at lower expression levels (Figure 16A), thus resembling the expression pattern of *Nolz1 *(Figure 2). To determine if *Nolz1 *and *Tle4 *were co-expressed in the LGE, we performed *in situ *hybridization for *Nolz1 *followed by immunohistochemistry for Tle4, which confirmed their colocalization within the LGE (Figure 16B). We also analyzed the *Tle4 *expression pattern during NPC differentiation. *Tle4 *followed a pattern of expression similar to that of *Nolz1*, as its maximal levels were achieved in non-differentiated NPCs and decreased with differentiation (Figure 16C).

## Discussion

Our results show that *Nolz1 *expression is high in the SVZ and is maintained at low levels in the MZ of the LGE, suggesting a dual effect promoting cell cycle exit and neuronal differentiation of NPCs. We found that *Nolz1 *expression negatively regulates the proliferation and induces cell cycle exit of NPCs cultured as neurospheres. In addition, in LGE-derived primary neural cultures, *Nolz1 *over-expression increased the number of neurons at the expense of nestin- and GFAP-positive cells, indicating that *Nolz1 *participates in striatal neurogenesis. We also show that *Nolz1-*induced neurogenesis depends on RA signaling, since this transcription factor promoted the expression of RARβ in LGE-derived NPCs and its neurogenic effect was impaired in a RA-signaling-free context.

The shape of the telencephalon and the relative sizes and positions of its cell populations result from variations in cell proliferation [37] and many transcription factors have important roles in this mechanism. Our results show that *Nolz1 *contributes to the homeostasis of NPCs in the SVZ of the vLGE, where it is highly expressed ([19] and our present results), by reducing the proliferation, promoting cell cycle exit and possibly reducing the self-renewal potential of NPCs. Cell cycle exit during LGE embryonic development is usually associated with proneural transcription factors. Here we demonstrate that *Nolz1 *promotes the acquisition of a neuronal phenotype since over-expression of *Nolz1 *in LGE primary cultures produces an increase in the number of neurons. Proneural factors such as *Ascl I *(also known as *Mash1*) [32] are restricted to the VZ and SVZ, where progenitors begin to differentiate, but are absent in the MZ, where fully differentiated neurons are found. However, other transcription factors such as *Meis2 *or *Islet *are expressed in both the germinal VZ and SVZ and in the MZ [28,38]. Similarly, *Nolz1 *expression is high in the SVZ and its expression decreases but does not disappear in the MZ, where striatal neurons continue to express low levels of *Nolz1 *([19] and present results). These results agree with the idea that stable specification of cell identity requires mechanisms that maintain patterns of gene expression over long periods of time [39].

One of the main issues in cell fate specification is the understanding of the interaction between two general sets of determinative factors: secreted extrinsic signals present in a cell's local environment and intrinsic signals that operate in a cell-autonomous manner [39]. Keeping with this view, RA signaling can increase *Nolz1 *levels in the PC12 cell line [19] and in chick postmitotic motoneurons [20]. Our results demonstrate that *Nolz1 *expression was not modified when a vitamin A-deficient diet was used to feed the mothers, which results in lowered levels of RA synthesis in the pups throughout the whole of development [31]. In addition, *Nolz1 *expression was not affected in Raldh3-deficient animals, which have been shown to lack RA activity in the LGE [11]. These findings suggest that the levels of RA are not limiting for *Nolz1 *expression. Reinforcing this idea, we observed that RA does not regulate *Nolz1 *expression in E14.5-derived NPCs or primary cultures. However, E12.5-derived NPCs do respond to RA by increasing the expression of *Nolz1*. Similarly, RA was able to regulate *Nolz1 *in mouse embryonic stem cells. Altogether, these data indicate that the ability of cells to induce *Nolz1 *in response to RA is dynamically regulated during striatal development. Thus, a very interesting possibility is that RA participates in the induction but not in the maintenance of *Nolz1 *expression, since other sources of RA in the LGE has been described [40].

Interestingly, we found that *Gsx2 *is required for the correct expression of *Nolz1 *in the vLGE, the mouse telencephalic structure that gives rise to striatal projection neurons (for a review, see [41]). In agreement with this result, it has recently been shown that *Gsx2 *participates in the temporal specification of neuronal fate in the LGE [42]. The development of striatal projection neurons depends on early actions of *Gsx2 *during differentiation of the vLGE, where *Nolz1 *is expressed, while differentiation of the dLGE, where *Nolz1 *is not expressed, requires *Gsx2 *function at least until birth [42]. However, over-expression of *Gsx2 *in NPCs did not modify the levels of *Nolz1*, suggesting that *Gsx2 *acts as a permissive instead of an inductive factor for *Nolz1 *expression. Since *Gsx2 *is involved in the differentiation of striatal projecting neurons through the regulation of RA signaling [18], *Nolz1 *could act downstream of *Gsx2 *to regulate RA signaling. We found that *Nolz1 *regulates the expression of RARβ, the RA receptor that has been shown to promote striatal projection neuron differentiation [12,15,43]. In addition, early striatal neurogenesis is affected in RARβ mutant mice [44], coinciding with the expression and function of *Nolz1*. In fact, *Nolz1 *and *RARβ *are expressed in the MZ at the same developmental stages ([19,45] and present results). Furthermore, our present results demonstrate that *Nolz1 *induces not only increases in *RARβ *but also RA signaling, which is essential to exert its neurogenic effect in primary striatal cultures. Interestingly, although this receptor is necessary for *Nolz1*-induced neurogenesis, its activation by a selective RARβ agonist is not sufficient to stimulate any changes in neural markers. This result suggests that *Nolz1 *may also act on additional pathways to promote neural differentiation. Altogether, these findings show that *Nolz1 *participates in the early *Gsx2*-dependent differentiation of vLGE-derived neurons through the regulation of RARβ-mediated signaling.

It has been shown that *Gsx2 *mutant mice have reduced levels of Raldh3 [18]. In addition, we show that over-expression of *Gsx2 *in NPCs increases the mRNA levels of this striatal RA-limiting enzyme. However, we observed that *Nolz1 *did not regulate the expression of Raldh3, so it is not regulating RA synthesis. These results suggest that *Gsx2 *may regulate RA signaling at two different levels; one is Nolz-independent and mediated by the regulation of Raldh3 and the other involves the regulation of *Nolz1 *expression, which in turn regulates the levels of *RARβ*.

*RARβ *is expressed in the MZ while *RARα *is broadly expressed in the VZ and SVZ through the entire telencephalon and is important in the control of the proliferation of neural precursors [11,46]. We observed that *RARα *is unaffected by *Nolz1 *over-expression. Thus, it seems that *Nolz1 *acts through RA signaling to regulate the differentiation of striatal projection neurons but its effect on NPC proliferation is independent of RA. In fact, we observed that RA did not modify the proliferation of NPCs *in vitro*. Increases in cell cycle length could lead to progressive restriction of the proliferation potential of LGE NPCs, and it can be promoted by transcription factors such as *Foxg1 *[37]. However, *Nolz1 *did not induce changes in cell cycle length. Another interesting possibility is that *Nolz1 *and *Gsx2 *function together to regulate proliferation since *Gsx2 *expression in the SVZ coincides with *Nolz1 *([19,32] and present results). Keeping with this view, it has been shown that the *Gsx2 *homolog in *Drosophila*, *Ind *(*intermediate neuroblasts defective*), interacts with Gro proteins, which are able to bind *elB *(*elbow B*), the *Nolz1 *homolog, acting as transcriptional co-repressors [27]. In addition, the mammalian *Nolz1 *protein also contains the conserved FKPY sequence, which allows binding to Gro-TLE proteins (Figure 1A). We observed that many TLE (NocA-*elB*-Tlp) proteins, the mammalian homologs of Gro, are expressed in the vLGE. Among them, TLE4 follows a similar expression pattern to Nolz1, with its highest levels in the SVZ, while the VZ is enriched in TLE1 expression. These results are similar to those observed in the cerebral cortex, where TLE4 is expressed by more differentiated NPCs of the SVZ and TLE1 expression is elevated in the undifferentiated VZ neural precursors [47-49]. Moreover, TLE4 expression decreased upon NPC differentiation. Taken together, these findings suggest that Nolz1 and TLE4 could act together to control SVZ proliferation in the LGE. Similarly, it has been recently described that Nolz1 requires, in part, the modulatory activity of Grg5, an atypical member of the Gro-TLE family of co-repressors, to control motor neuron determination in the chick spinal cord [20]. However, further experiments are required to analyze functional interactions between these proteins.

In conclusion, we demonstrate that *Nolz1 *has a dual effect on NPCs, on one hand by controlling their proliferation and promoting cell cycle exit, and on the other by inducing striatal neurogenesis. *Nolz1 *over-expression increases the number of striatal neurons downstream of *Gsx2 *by inducing RA signaling through RARs. Its properties and expression pattern suggest that the activity of Nolz1 in the vLGE could be modulated by co-repressors, such as Gro-TLE.

## Materials and methods

### Animal subjects

All animals were housed with access to food and water *ad libitum *in a colony room maintained at a constant temperature (19 to 22°C) and humidity (40 to 50%) on a 12:12 h light:dark cycle. Animal treatments and handling procedures were approved by the Local Committees, in accordance with the European Community Council Directive (86/609/EU).

B6CBA wild-type mice (from Charles River Laboratories, Les Oncins, France), Raldh3-deficient mice [11], and *Gsx2*-deficient mice [32] were used in this study. For embryonic ages, time of pregnancy was determined by first detection of a vaginal sperm plug by daily inspection and considered as E0.5. For postnatal studies, the day of birth was considered as postnatal day 0 (P0).

To induce vitamin A deficiency in mice, pregnant mice were fed with the vitamin A-deficient diet TD.86143 (Harlan Laboratories Inc., Indianapolis, IN, USA).

### Culture procedures

E12.5 or E14.5 fetal brains were excised and placed in sterile phosphate-buffered saline pH 7.4, and the LGEs were dissected bilaterally, pooled and gently dissociated with a fire-polished Pasteur pipette.

Mixed neuron-glial primary cultures were obtained by plating the cells onto 24-well plates containing glass coverslips precoated with 0.1 mg/ml poly-D-lysine (Sigma Chemical Co., St Louis, MO, USA) at a density of 150,000 cells/cm^2 ^in Eagle's minimum essential medium (Invitrogen SA, Prat de Llobregat, Barcelona, Spain) supplemented with 7.5% fetal bovine serum (FBS; Invitrogen SA), 0.6% D-(+)-glucose (Sigma Chemical Co.), 100 U/mL of penicillin and 100 mg/mL streptomycin (both obtained from Invitrogen SA). Three or 5 days after seeding, cultures were fixed with 4% paraformaldehyde solution (PFA; Merck Biosciences Ltd, Nottingham, UK) in 0.1 M phosphate buffer pH 7.4 and processed for immunocytochemistry.

LGE-derived neurosphere cultures were obtained by seeding 50,000 cells/cm^2 ^in medium containing Dubelcco's Modified Eagle's Medium (DMEM; Sigma Chemical Co.):F12 (Invitrogen SA) (1:1); supplemented with 0.3% glucose (Sigma Chemical Co.), 0.3 mg/ml glutamine (Invitrogen SA), 5 mM HEPES (Invitrogen SA), 100 U/ml penicillin, and 100 mg/ml streptomycin (Invitrogen SA), 4 μg/ml heparin (Sigma Chemical Co.), 4 mg/ml bovine serum albumin (Sigma Chemical Co.), 1× N2 supplement (Invitrogen SA), 20 ng/ml fibroblast growth factor (Sigma Chemical Co.) and 10 ng/ml epidermal growth factor (Invitrogen SA). Every 5 days neurospheres were collected, dissociated by pipetting approximately 40 times with a P100 micropipette and re-plated in fresh media at a density of 10,000 cells/cm^2^.

For cell differentiation, 12,500 cells/cm^2 ^were seeded. The day after plating, cells were collected and incubated onto Matrigel-treated coverslips (Growth Factor Reduced Matrigel Matrix, BD Biosciences, Clontech-Takara Bio Europe, Saint-Germain-en-Laye, France). Media was changed to medium supplemented with only 20 ng/ml fibroblast growth factor (Sigma Chemical Co.) and cells were allowed to differentiate in this medium for 2 more days. Then, media was changed again to medium supplemented with 2% FBS (Invitrogen SA) and cells were grown for 3 more days (until a total of 6 days of *in vitro *differentiation). Cell pellets for each time point (0, 3 and 6 days of differentiation) were obtained and frozen at -80°C for RNA or protein extraction.

In the present study, we used the mouse embryonic stem cell line R1 obtained from Dr Andras Nagy's laboratory. The maintenance of undifferentiated mouse embryonic stemcells, embryoid body formation and culture were carried out as previously described [15].

All cell cultures were incubated at 37°C in a 5% CO_2 _atmosphere.

### RA and RARβ agonist treatment

Neurospheres were passaged as described above and single cells were seeded in 6-well plates at a density of 100 cells/mm^2 ^with fresh culture medium containing different concentrations of all-trans-RA (10^-9^, 10^-8 ^and 10^-6 ^M; Sigma Chemical Co.) dissolved in dimethyl sulfoxide (DMSO; Sigma Chemical Co.). Control cells were cultured with the same dilutions of the RA vehicle, DMSO. Some cultures were treated with a RARβ-specific agonist [36] at a concentration of 10^-8 ^M dissolved in DMSO, which we observed is effective in these cultures (R Martín-Ibáñez *et al*., in preparation). NPCs were allowed to grow for 3 DIV and then were pelleted for RNA extraction and RT-PCR analysis or processed for BrdU immunocytochemistry.

Mixed neuron-glial LGE primary cultures were grown for 3 DIV and RA or RARβ agonist (BMS641) dissolved in DMSO was added to the medium at the concentration of 10^-8 ^M. Fresh RA/RARβ agonist was added every 24 hours of culture and 3 hours prior to fixation.

Embryoid bodies were formed as described above and RA was added to the culture medium at different concentrations (10^-9^, 10^-8 ^and 10^-6 ^M). They were grown for 4 DIV and then pelleted for RNA extraction and RT-PCR analysis.

### Cell transfection

To over-express *NolzI*, cells were transfected with the pNolz-IRES2-DsRED plasmid, that was obtained by clonation of the human *NolzI *contained in the pOTB plasmid (MGC full-length (IRAU) collection, clone ID 4053098) into EcoRI and SmaI restriction sites of p-IRES2-DsRED-Express plasmid (BD Biosciences) coding for DsRED fluorescent protein. As a control, we used pIRES2-DsRED-Express empty plasmid. Tranfection was made with 9 μg of NolzI-RED or RED plasmid.

To reduce *NolzI *expression, three different siRNAs against *NolzI *mRNA were used (Silencer Pre-designed siRNAs, IDs 89661, 169777, 89565, Ambion, Applied Biosystems, Foster City, CA, USA). Tranfection was made with 2 μM of each siRNA or 6 μM of negative control siRNA (Silencer Negative Control #1 siRNA, Ambion).

Low passage (four to seven) embryonic neurospheres were disaggregated and transfected by nucleofection following the manufacturer's protocol (Amaxa Biosystems, Lonza Iberica SA, Barcelona, Spain). Using the A33 Nucleofector program (Amaxa Biosystems), 5 × 10^6 ^cells were transfected. Viable cells were counted by trypan blue exclusion in a Neubauer chamber after nucleofection.

For BrdU incorporation assays, 65,000 cells/cm^2 ^were seeded after nucleofection. Two days later cells were collected and incubated for 10 minutes in 24-well plates with Matrigel-treated coverslips and they were incubated for 10 minutes in BrdU-containing media at a final concentration of 2 μg/ml. Just after incubation, cells were fixed and processed for immunocytochemistry.

The cell cycle exit index was analyzed as described elsewhere [30]. Briefly, we performed the same procedure as for BrdU incorporation except that neurospheres were pulsed with BrdU the same initial day of inducing *hNolz *over-expression and cells were fixed 3 days later. Thereafter, neurosphere cultures were processed for BrdU and Ki67 immunostaining. BrdU-positive but Ki67-negative cells were counted as the cells that left the cell cycle during the experimental period.

In order to analyze cell cycle duration, 10 neurospheres were plated 3 days after transfection in 96-well plates with complete medium and supplemented with 1 μM BrdU. Neurospheres were attached at 1, 3, 6, 12 and 24 hours after treatment in 96 well-plates pre-coated with Matrigel. Ten minutes later, cells were fixed in 4% PFA and processed for immunocytochemistry.

For the self-renewal assay, 65,000 cells/cm^2 ^were seeded after nucleofection and the total number of neurospheres obtained 5 days later was counted (passage 0 (P0) after transfection). Cells were dissociated and 2,500 cells/cm^2 ^were seeded and counted again on day 5 (passage 1 (P1) after transfection).

For differentiation after nucleofection, 50,000 cells/cm^2 ^were seeded and the same protocol as for non-nucleofected cells was followed.

To over-express *Nolz1 *in primary cultures, we transfected the cells with the pLV-*Nolz*-IRES-EGFP plasmid or the pLV-IRES-EGFP plasmid, which encode *hNolz *and EGFP or EGFP only, respectively. The pLV-IRES-EGFP plasmid was generated using the pRRLsinPPT plasmid (pRRL) constructed by the Miami Project to Cure Paralysis Viral Vector Core Lab based on the lentiviral transducing plasmid developed by Naldini *et al*. [50]. Briefly, the multiple cloning site (MCS) of the pRRL plasmid was substituted by the MCS-IRES-EGFP from the PRV-IRES-EGFP (Genetrix SL, Tres Cantos, Madrid, Spain) using the BamHI and the SalI restriction sites. To construct the pLV-*Nolz*-IRES-EGFP, the *hNolz *gene from the p*Nolz*-IRES2-DsRED plasmid was cloned into pLV-IRES-EGFP between the MCS BamHI and XhoI sites. Primary cultures were transfected 24 hours after seeding with 0.5 μg of the corresponding plasmids per well (24-well plate). The transfection was performed using Lipofectamine LTX (Invitrogen SA), following the manufacturer's instructions. Three days after transfection cells were fixed with 4% PFA for immunocytochemistry analysis.

For DEAB (Sigma Chemical Co.) treatment, primary cultures were transfected 12 h after seeding as described above. Then, 24 h later DEAB was added to the medium at a concentration of 10^-8 ^M. Three days after treatment cells were fixed with 4% PFA for immunocytochemistry analysis.

For RAR inverse agonist administration, primary cultures were transfected 12 h after seeding as described above. Thereafter, the RAR inverse agonist (BMS493 [35]) was added to the medium at a concentration of 10^-8 ^M in DMSO, which was repeated every single day. At 3 DIV cells were fixed with 4% PFA for immunocytochemistry analysis.

### Production of viral particles and cell transduction

To over-express *Gsx2*, the human *Gsx2 *gene from the pcDNA-hGsx2 plasmid, kindly provided by Dr Peter Marynen (Université de Leuven, Belgium), was PCR-cloned into the retroviral vector pRV-IRES-EGFP using the MCS BamHI and XhoI sites.

For retrovirus production, 293T cells were plated at a density of approximately 6 × 10^4 ^cells per cm^2^. The following day, cells were transfected by a three-plasmid system (the pRV-Gsx2-IRES-GFP plasmid, the plasmid that expresses HIV-1 *gag *and *pol *genes, and the plasmid that expresses vesicular stomatitis virus G) using the calcium phosphate/DNA co-precipitate method. The transfection mixture remained on the cells for 7 h before the transfection medium was replaced with fresh medium. The supernatant from vector-producing 293T cells was recovered every 22 h during 3 days before being harvested, passed through a 0.45-μm-pore-size filter to remove producer cells, and then subjected to two centrifugations at 4°C and 22,000 × *g *for 90 minutes to concentrate the virus. The virus-containing pellet was dissolved in 1% bovine serum albumin. Viral concentrate (20 μl) from pRV-Gsx2-IRES-EGFP or pRV-IRES-EGFP was used to transduce 3.5 × 10^6 ^dissociated NPCs in a 6-well plate as described previously [51]. Transduced NPCs were growth as neurospheres as described above for 5 DIV before being pelleted for RNA extraction.

### Generation of the anti-Nolz1 antibody

Anti-Nolz1 polyclonal antibodies were obtained from the serum of immunized rabbits with a keyhole limpet hemocyanin (KLH)-conjugated oligopeptide coding for amino acids 2 to 14 of the Nolz1 sequence (MSTAPSLSALRSSKH; Figure 1A). Pre-immune serum was obtained from the same rabbits before immunization.

### Immunolabeling

All immunostaining was performed using the following antibodies: polyclonal anti-*NolzI *(1:10,000), monoclonal anti-GFAP (1:500; Sigma Chemical Co.), monoclonal anti-Tuj1 (1:500; Sigma Chemical Co.), monoclonal anti-BrdU (1:50; Dako A/S, Glostrup, Denmark), monoclonal anti-MAP2 (1:200; Sternberger Monoclonals, Lutherville, MD, USA), polyclonal anti-nestin (Rat 401; 1:40; Developmental Studies Hybridoma Bank; The University of Iowa, Iowa), polyclonal anti-Tle4 (1:200; generous gift from Dr Stefano Stifani, McGill University), polyclonal conjugated FITC-GFP (1:200; ABCAM, Cambridge, UK), polyclonal anti-Ki67 (1:200; Thermo Fisher Scientific SLU, Alcobendas, Madrid, Spain). For Nolz negative controls, pre-immunization serum was used at the same concentration as NolzI antibody-containing serum. Preparations were counterstained with DAPI to visualize the nucleus.

No signal was detected in control immunostaining assays in which the primary antibody was omitted.

### *In situ *hybridization

We analyzed the expression of several genes by radioactive *in situ *hybridization as described elsewhere [52]. The following oligonucleotide probes were used: mouse *NolzI *- complementary to nucleotides 3,226 to 3,266 of the *NolzI *sequence (GenBank accession number NM_145459); mouse *Tle1 *- complementary to nucleotides 1,942 to 1,983 of the *Tle1 *sequence (GenBank accession number NM_011599); mouse *Tle2 *- complementary to nucleotides 1,351 to 1,389 of the *Tle2 *sequence (GenBank accession number NM_019725); mouse *Tle3 *- complementary to nucleotides 3,434 to 3,474 of the *Tle3 *sequence (GenBank accession number NM_001083927); mouse *Tle4 *- complementary to nucleotides 1,614 to 1,652 of the *Tle4 *sequence (GenBank accession number NM_0011600).

### Quantitative PCR assays

Expression of several genes was evaluated by Q-PCR assays performed as previously described [15], using the following TaqMan^® ^gene expression assays (Applied Biosystems): 18S, Hs99999901_s1; nestin, Mm00450205_m1; β-tubulin III, Mm00727586_s1; GFAP, Mm00546086_m1; MAP2, Mm00485230_m1; Gsx2, Mm00446650_m1; mouse *NolzI*, Mm00520908_m1; Raldh3, Mm00474049_m1; RARα, Mm00436264_m1; RARβ, Mm01319674_m1; RARγ, Mm00441083_m1; CRBP1, Mm00441119_m1; Cyp26b1, Mm00558507_m1; Tle4, Mm01195160_m1. To specifically recognize *hNolz*, a customized Taqman^® ^Assay was designed consisting of the following primers: forward, CCTCGCCCTCCTCCAAAC; reverse, GCCCGATTTGGTGTCCTTGT; reporter, TCTCCTCGGTTGCCTCC. To provide negative controls and exclude contamination by genomic DNA, the reverse transcriptase was omitted in the cDNA synthesis step, and the samples were subjected to the PCR reaction with each TaqMan^® ^gene expression assay.

Analysis and quantification was performed with the Comparative Quantitation Analysis program of the MxPro™ Q-PCR analysis software version 3.0 (Stratagene, La Jolla, CA, USA), using the 18S gene expression as internal loading control. All Q-PCR assays were performed in duplicate and repeated for at least three independent experiments. The results were expressed as relative levels with respect to the expression of the same gene in the control condition, considered as 100%.

### Western blotting

We analyzed the levels of Nolz1 protein in transfected neurospheres or in the striatum at different developmental stages. Samples (at least n = 3 per time point) were prepared and processed for western blotting as described elsewhere [53]. Blots were incubated overnight at 4°C with anti-NolzI antibody (1:50,000). The secondary antibody was a horse radish peroxidase-conjugated anti-rabbit IgG (1:3,000; Promega Biotech Iberica, SL., Madrid, Spain) and the signal was developed using the ECL western blotting analysis system (GE Healthcare Europe GMBH, Cerdanyola del Vallès, Barcelona, Spain).

### Cell counts

In order to determine the role of *NolzI *on the proliferation of progenitor cells *in vitro*, we counted the number of cells that incorporate BrdU. BrdU- and EGFP-positive (transfected) cells were detected by immunocytochemistry and the total number of cells determined by DAPI counterstaining. For over-expressing experiments the results were expressed as the percentage of proliferating cells with respect to the transfected (EGFP-positive) cells, while for siRNA experiments, the results were expressed as the percentage of proliferating cells with respect the total number of cells (n = 5). We also counted by phase contrast the total number of neurospheres 5 days after *hNolz *or control transfection.

Cell death was evaluated by counting the number of apoptotic nuclei stained with DAPI after *hNolz *over-expression. The results were expressed as the percentage of dying cells with respect to transfected (EGFP-positive) cells (n = 4). Results were normalized with respect to control-transfected NSCs (considered as 100%).

We estimated the cell cycle time as previously described [1,29]. Briefly, BrdU was added to neurosphere cultures during the last 1, 3, 6, 12 and 24 h of culturing. The number of BrdU-positive cells and the total number of cells, determined by DAPI counterstained nuclei, in each neurosphere was counted. The percentage of proliferating cells was calculated for each time point after BrdU administration. Regression analyses of the active portion of each BrdU labeling curve were used to estimate cell cycle time assuming that all cells proliferate at the same rate and that every cell is labeled at the end of a single cycle [29]. The r^2 ^of this lineal correlation was used to calculate the cell cycle duration. We counted at least 30 neurospheres in each condition in 3 transfected cultures.

We analyzed the cell cycle index as the number of cells that retain BrdU but leave the cell cycle (Ki67-negative cells) after a 3-DIV pulse label. Thus, we counted the fraction of BrdU+/Ki67- cells and normalized to the total number of BrdU-positive cells in the culture. Results were expressed as absolute percentages in each condition.

To determine the effect of *Nolz1 *on the differentiation of LGE primary cultures, we counted the number of cells per coverslip overexpressing *hNolz *or EGFP that colocalized with different markers, such as nestin, Tuj1 and MAP2 3 or 5 days after the transfection. The results are expressed as the percentage of transfected cells colocalizing with the different markers with respect to the total number of transfected cells. Between 50 and 200 transfected cells per coverslip were counted per transfection (n = 3 to 5).

### Brain slice electroporation

Coronal brain slices (250 μm) from E14.5 mice embryos were obtained with a vibratome. Slices were plated onto culture membranes with minimum essential medium supplemented with 10% FBS and 50 U/ml penicillin-streptomycin. After 1 hour in the incubator, media was changed to Neurobasal supplemented with B27, 1 mM HEPES, 50 U/ml penicillin-streptomycin and 2 mM L-glutamine. Two hours later, slices were electroporated with 8 μg of DsRED-*Nolz *or DsRED plasmid. After 48 hours in culture, electroporated slices were fixed during 2 hours with 4% PFA, dehydrated with increasing ethanol concentrations and stored until processing for immunohistochemistry.

We counted the number of Ki67-positive cells present in the electroporated zone of brain slices. First, we took a picture of the slices showing the RED fluorescence of the electroporated plasmids prior to fixation. Then, the slices were immunostained against Ki67 and the number of positive cells included in the electroporated area for the control side (DsRED plasmid) or experimental side (*Nolz1*-DsRED) were counted. The area to count was fixed by delineating the electroporated DsRED positive area in the pre-immunostaining image using ImageJ, and transferring this area to the Ki67 stained image. At least four different slices were counted for each condition.

### Luciferase RARE reporter assay

To monitor the RARE activity we used the Cignal RARE Reporter Assay Kit (SABioscience Corporation, Frederick, MD, USA) following the manufacturer's instructions. Mixed neuron-glial LGE primary cultures were performed as described above and 24 hours after seeding cells were transfected with the inducible RARE-responsive mixture and the pLV-*Nolz*-IRES-EGFP or the pLV-IRES-EGFP plasmids. The transfection was performed using Lipofectamine LTX (Invitrogen SA) following the manufacturer's instructions. Two days later, cultures were processed to evaluate luciferase using the Dual-Luciferase Reporter Assay System (Promega). The firefly/*Renilla *luciferase ratio was calculated for each well, and results are expressed as the mean of four independent experiments and normalized with respect to control-transfected primary cultures (considered as 100%).

### Statistical analyses

All results are expressed as the mean of independent experiments ± standard error of the mean. Results were analyzed using the Student's *t*-test or one-way ANOVA followed by the Bonferroni *post-hoc *test.

## Abbreviations

BrdU: bromodeoxyuridine; DEAB: 4-diethylaminobenzaldehyde; DIV: days *in vitro*; dLGE: dorsal LGE; DMSO: dimethyl sulfoxide; E: embryonic day; EGFP: enhanced green fluorescent protein; elB: elbow B; FBS: fetal bovine serum; GFAP: glial fibrillary acidic protein; GFP: green fluorescent protein; Gro: Groucho; hNolz: human Nolz; LGE: lateral ganglionic eminence; MAP: microtubule-associated protein; MCS: multiple cloning site; MZ: mantle zone; NET: NocA-Elbow (*elB*)-Tlp; NPC: neural progenitor cell; PFA: paraformaldehyde; Q-PCR: quantitative PCR; RA: retinoic acid; Raldh: retinaldhehyde dehydrogenase; RAR: RA receptor; RARE: RA response element; siRNA: small interfering RNA; SVZ: subventricular zone; TLE: NocA-*elB*-Tlp; Tuj1: β-III tubulin; vLGE: ventral LGE; VZ: ventricular zone.

## Competing interests

The authors declare that they have no competing interests.

## Authors' contributions

NU, collection and assembly of data, data analysis and interpretation, manuscript writing, final approval of manuscript; RM-I, CH, ME, EC, MP, HRM-G, RW, CC, SA, RA, collection and assembly of data, final approval of manuscript; GD, KC, ARdL, CV-A, SM, JA, financial support, data analysis and interpretation, final approval of manuscript; JMC, conception and design, financial support, administrative support, data analysis and interpretation, manuscript writing, final approval of manuscript.
